# The Relationship Between Serum Concentration of Vitamin D, Total Intracranial Volume, and Severity of Depressive Symptoms in Patients With Major Depressive Disorder

**DOI:** 10.3389/fpsyt.2019.00322

**Published:** 2019-05-09

**Authors:** Dao-min Zhu, Wenming Zhao, Biao Zhang, Yu Zhang, Ying Yang, Cun Zhang, Yajun Wang, Jiajia Zhu, Yongqiang Yu

**Affiliations:** ^1^Department of Sleep Disorders, Hefei Fourth People’s Hospital, Hefei, China; ^2^Anhui Mental Health Center, Hefei, China; ^3^Department of Radiology, The First Affiliated Hospital of Anhui Medical University, Hefei, China

**Keywords:** major depressive disorder, magnetic resonance imaging, vitamin D, total intracranial volume, mediator

## Abstract

**Background:** Depression has been linked to vitamin D deficiency. However, little attention was paid to the neural substrate underlying this association.

**Methods:** Fifty patients with major depressive disorder (MDD) were enrolled in this study. High-resolution structural magnetic resonance imaging was performed to calculate total intracranial volume (TIV). Peripheral venous blood samples were collected to measure serum vitamin D concentration. Hamilton Rating Scale for Depression (HAMD) was used to assess severity of depression symptoms. The relationship among TIV, serum vitamin D concentration, and HAMD score was examined using correlation, linear regression, and mediation analyses.

**Results:** In patients with MDD, HAMD score was negatively correlated with TIV and serum vitamin D concentration, and TIV was positively correlated with serum vitamin D concentration. Linear regression analyses showed that TIV and serum vitamin D concentration were significant predictors of HAMD score. Importantly, mediation analysis revealed that TIV significantly mediated the relationship between serum vitamin D concentration and HAMD score.

**Conclusion:** Our findings suggest that TIV may serve as a potential neural biomarker for monitoring responses to adjuvant therapy of vitamin D in patients with MDD.

## Introduction

Major depressive disorder (MDD) is a severe mental disorder, which diminishes the functioning and quality of life (1) and leads to a heavy social and economic burden (2). A recent systematic review and network meta-analysis has found that almost all antidepressants are more efficacious than placebo in adults with MDD and thus antidepressants are used more frequently than other interventions (3). Another network meta-analysis has found that although fluoxetine might reduce depressive symptoms in children and adolescents with MDD, most antidepressants do not seem to be suitable as routine treatment options for MDD in young people because of poor efficacy and tolerability (4). The strength and limitation of antidepressants’ action are suggestive of other modifiable risk factors that have not been fully considered but can be targeted to prevent and treat depression. For example, there is evidence that some nutritional components (5), such as vitamin D, might play an important role in mental health and neuropsychiatry disorders including MDD (6–9).

Vitamin D consists of two forms: ergocalciferol (vitamin D_2_) and cholecalciferol (vitamin D_3_). Vitamin D_2_ is present in plants and some types of fish, and vitamin D3 is produced when the skin is exposed to ultraviolet B (UVB) rays from sunlight (10). It is estimated that 12 min of midday exposure to the sun without sunscreen is equivalent to 3,000 IU of vitamin D a day intake of the diet (11). 25-Hydroxyvitamin D [25(OH)D] is the main circulating metabolite of vitamin D and the serum 25(OH)D level is the most reliable marker of vitamin D status (12). The discovery of vitamin D metabolites in cerebrospinal fluid in healthy adults suggests that vitamin D may play a role in brain development (13). Furthermore, the identification of vitamin D receptor (VDR) in the human central nervous system (CNS) indicates that vitamin D may have a functional role in the nervous system (14, 15). VDR is expressed in the prefrontal cortex, cingulate cortex, basal forebrain, amygdala, caudate/putamen, thalamus, substantia nigra, lateral geniculate nuclei, hypothalamus, cerebellum, and hippocampus (16, 17). VDR expression in the prefrontal cortex and limbic system illustrates the role of vitamin D in maintaining affect, emotional, and cognitive functions (14, 18, 19). Over the past decade, there is increasing interest in the interaction between vitamin D deficiency and the development or clinical manifestations of psychiatric disorders, especially depression. Several review and meta-analysis literatures have suggested that depression is linked to vitamin D deficiency (20, 21) and that vitamin D supplements are beneficial for the treatment of depression (22, 23). However, the neural substrate underlying the association between vitamin D and depression remains indiscernible.

Prior neuroimaging studies have demonstrated that serum vitamin D level alterations are associated with brain volume changes (24–27) and patients with MDD have widespread cortical and subcortical volume abnormalities (28–34). Given these findings, one may speculate that there is an association among serum vitamin D concentration, severity of depressive symptoms, and total intracranial volume (TIV, a global and unbiased volumetric parameter) in patients with MDD. In this study, we aimed to clarify this issue and hypothesized that TIV would mediate the relationship between serum vitamin D concentration and severity of depressive symptoms.

## Materials and Methods

### Participants

We consecutively recruited patients from the outpatient and inpatient departments of Hefei Fourth People’s Hospital. A total of 50 right-handed patients with MDD were comprised in this study. Two well-trained clinical psychiatrists confirmed the diagnoses of depression in accordance with the International Classification of Diseases (ICD-10) criteria. Exclusion criteria for all participants included 1) the presence of other psychiatric disorders such as bipolar disorder, schizophrenia, anxiety disorders, substance-induced mood disorder, substance abuse, or dependence; 2) a history of significant physical or neurological diseases; 3) a history of head injury with loss of consciousness; and 4) contraindications for MRI (such as pregnancy). The severity of depression and anxiety symptoms was captured by using the 24-item Hamilton Rating Scale for Depression (HAMD) (35) and the 14-item Hamilton Rating Scale for Anxiety (HAMA) (36). All patients were receiving their regular antidepressant medications (see **Table S1** in the **Supplementary Materials**). However, combination therapies were frequently used and there was a considerable variability in the administered drugs and the current medication duration, which prevents us from testing differences between patients with distinct medication regimens. This more natural setting allows us to perform a more conservative analysis to identify aberrations in patients with MDD and thus should better reflect the overall population of patients with MDD. This study was approved by the ethics committee of The First Affiliated Hospital of Anhui Medical University. All participants provided written informed consent after they had been given a complete description of the study.

### Image Acquisition

MRI scans were acquired using a 3.0-T MR system (Discovery MR750w, General Electric, Milwaukee, WI, USA) with a 24-channel head coil. Earplugs were used to reduce scanner noise, and tight but comfortable foam padding was used to minimize head motion. During the scans, all participants were instructed to keep their eyes closed, relax but not fall asleep, think of nothing in particular, and move as little as possible. High-resolution 3D T1-weighted structural images were acquired by employing a brain volume (BRAVO) sequence with the following parameters: repetition time (TR) = 8.5 ms; echo time (TE) = 3.2 ms; inversion time (TI) = 450 ms; flip angle (FA) = 12°; field of view (FOV) = 256 mm × 256 mm; matrix size = 256 × 256; slice thickness = 1 mm, no gap; 188 sagittal slices; and acquisition time = 296 s.

### Image Processing

Voxel-based morphometry (VBM) analysis was performed using the CAT12 toolbox (http://www.neuro.uni-jena.de/cat) implemented in the Statistical Parametric Mapping software (SPM12, http://www.fil.ion.ucl.ac.uk/spm). First, all the structural T1-weighted images were corrected for bias-field inhomogeneities. Second, these images were segmented into gray matter, white matter, and cerebrospinal fluid using the “new-segment” approach. Finally, TIV was calculated as the sum of gray matter, white matter, and cerebrospinal fluid volumes.

### Serum Vitamin D Concentration Measurement

After an overnight fasting period, peripheral venous blood samples (2 ml) were collected from all patients in the morning of MRI scanning. Samples were sent to the Department of Clinical Laboratory, Hefei Fourth People’s Hospital immediately for centrifugation and serum was separated. Vitamin D [25(OH)D] was measured in serum using a chemiluminescence immunoassay (CLIA) technique in a fully automated Maglumi 1000 analyzer (SNIBE Co., Ltd., China). Internal quality control provided by the manufacturer was used to assure quality. Vitamin D level was stratified as follows: 30–100 ng/ml (75–250 nmol/L) as sufficiency, 20–29 ng/ml (50–72.5 nmol/L) as insufficiency, <20 ng/ml (50 nmol/L) as deficiency (37).

### Statistical Analysis

The statistical analyses were performed by using the SPSS 23.0 software package (SPSS, Chicago, IL). Pearson’s correlation analyses were performed to examine the associations among TIV, serum concentration of vitamin D, and HAMD score in the patients. Linear regression analyses were used to assess the predictive values of serum vitamin D concentration and TIV for HAMD score. In addition, to test whether the association between variables was mediated by other variables, mediation analysis was performed using the PROCESS macro (http://www.processmacro.org/) developed by Hayes (38, 39), a versatile modeling tool freely available for SPSS. The PROCESS uses an ordinary least squares path analytic framework to estimate direct and indirect mediation effects. In the mediation analysis model, all paths were reported as unstandardized ordinary least squares regression coefficients, namely, total effect of *X* on *Y* (*c*) = indirect effect of *X* on *Y* through *M* (*a* × *b*) + direct effect of *X* on *Y* (*c*′). The significance analysis was based on 5,000 bootstrap realizations, and the significance of indirect effects was assessed by bootstrap 95% confidence interval (CI). In the PROCESS analysis, a significant indirect effect is indicated when the bootstrap 95% CI does not include zero. In this study, only variables that showed a significant correlation with others in the correlation analyses were considered independent, dependent, or mediating variables in the mediation analysis. Finally, we divided the patients into two subgroups (patients with non-severe depressive symptoms: HAMD score < 24; patients with severe depressive symptoms: HAMD score ≥ 24) (40) and tested differences in serum vitamin D concentration and TIV between them. For these analyses, *P* < 0.05 was considered to indicate statistical significance.

## Results

### Participant Characteristics

The characteristics of participants are shown in **Table 1**. The mean age was 42.10 years (SD: 10.52 years) and 62% of the participants were females. The mean educational level was 9.30 years (SD: 3.80 years); HAMD, 29.42 (SD: 11.59); HAMA, 19.06 (SD: 7.41); illness duration, 66.33 months (SD: 74.66 months); onset age, 36.68 years (SD: 10.80 years); episode number, 2.5 (SD: 1.62); and TIV, 1,458.00 cm^3^ (SD: 157.90 cm^3^). Serum concentration of vitamin D was available for 45 of 50 patients with a mean value of 44.37 nmol/L (SD: 11.47 nmol/L). Moreover, among the 45 patients, 15 (33.33%) were classified as insufficiency and 30 were classified (66.67%) as deficiency.

**Table 1:** Summary of demographic and clinical characteristics of the sample.

Characteristics	Mean (SD)	Median	Range
Gender (female/male)	31/19	—	—
Age (years)	42.10 (10.52)	44.0	18.0–60.0
Education (years)	9.30 (3.80)	9.0	5.0–16.0
HAMD	29.42 (11.59)	28.0	3.0–52.0
HAMA	19.06 (7.41)	19.0	7.0–35.0
SCVD (nmol/L)*	44.37 (11.47)	43.49	20.47–66.59
Illness duration (months)	66.33 (74.66)	44.0	0.30–306.0
Onset age (years)	36.68 (10.80)	35.50	12.0–59.0
Episode number	2.50 (1.62)	2.0	1.0–10.0
TIV (cm³)	1,458.00 ± 157.90	1,415.24	1,131.41–1,739.80

HAMD, Hamilton Rating Scale for Depression; HAMA, Hamilton Rating Scale for Anxiety; SCVD, serum concentration of vitamin D; TIV, total intracranial volume.

*The data are available for 45 of 50 patients.

### Correlation and Linear Regression Analyses

**Figure 1:**
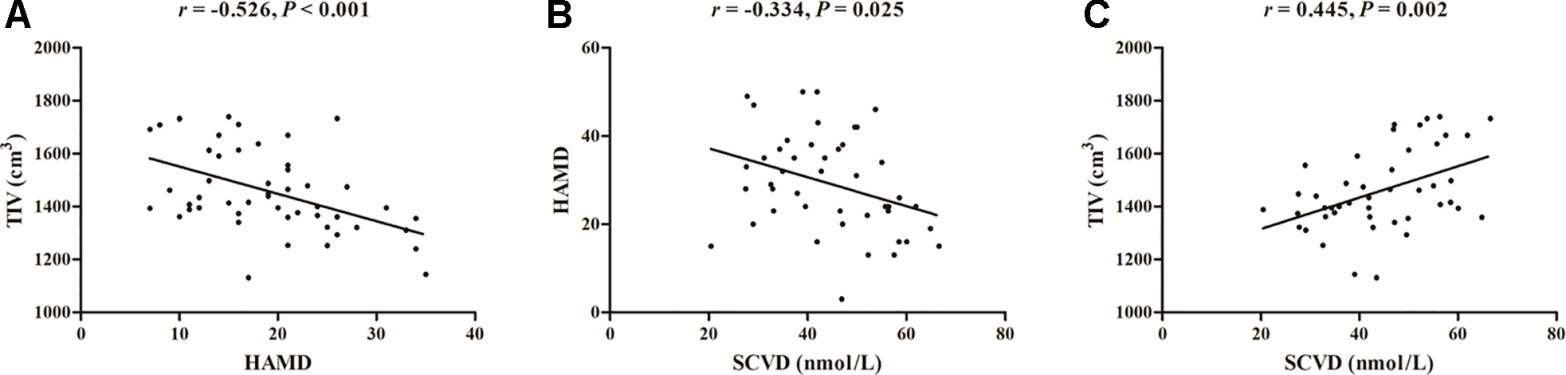
Correlations among TIV, HAMD, and SCVD in patients with major depressive disorder. **(A)** Scatter plot of the association between TIV and HAMD. **(B)** Scatter plot of the association between SCVD and HAMD. **(C)** Scatter plot of the association between SCVD and TIV. Abbreviations: TIV, total intracranial volume; HAMD, Hamilton Rating Scale for Depression; SCVD, serum concentration of vitamin D.

There were significant negative correlations between HAMD score and TIV (*r* = −0.526, *P* < 0.001; **Figure 1A**) and between HAMD score and serum vitamin D concentration (*r* = −0.334, *P* = 0.025; **Figure 1B**) in the patients with MDD. In addition, there was a positive correlation between TIV and serum vitamin D concentration (*r* = 0.445, *P* = 0.002; **Figure 1C**) in the patient group. Linear regression analyses showed that TIV (β = −0.039, *t* = −4.282, *P* < 0.001, *R* ^2^ = 0.276) and serum concentration of vitamin D (β = −0.328, *t* = −2.322, *P* = 0.025, *R* ^2^ = 0.111) were significant predictors of HAMD score, respectively. When TIV and serum concentration of vitamin D were included in a linear regression model (*R* ^2^ = 0.238), TIV remained predictive of HAMD score (β = −0.029, *t* = −2.640, *P* = 0.012), but serum concentration of vitamin D was not a significant predictor (β = −0.154, *t* = −1.043, *P* = 0.303).

### Mediation Analysis

**Figure 2:**
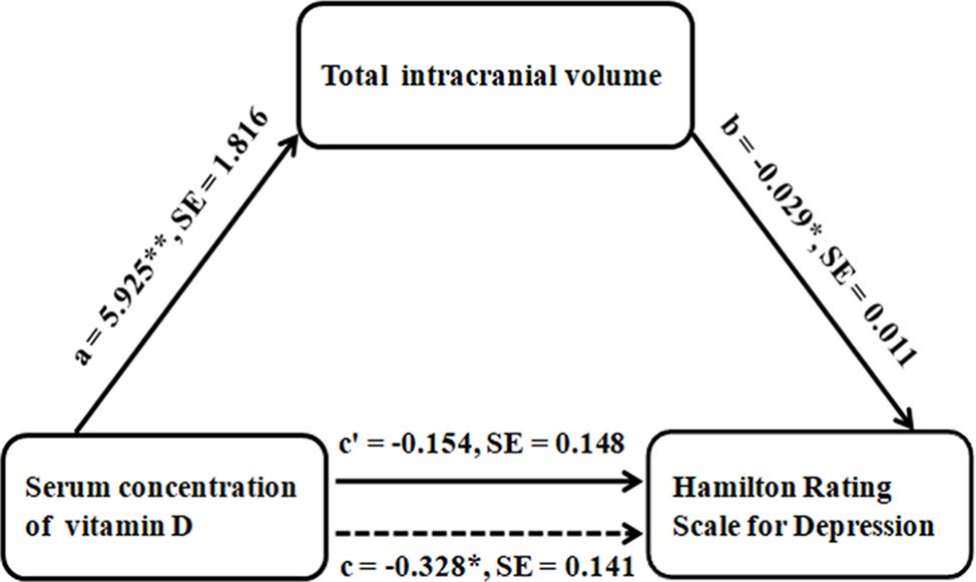
Graphical representation of the mediation analysis between SCVD and HAMD, with TIV as the mediator: estimates of the mediated, direct, and total effects. All paths are reported as unstandardized ordinary least squares regression coefficients. *P < 0.05, **P < 0.005. Abbreviations: SCVD, serum concentration of vitamin D; HAMD, Hamilton Rating Scale for Depression; TIV, total intracranial volume; SE, standard error.

The mediation analysis model and the investigated variables are described in **Figure 2**. There was a significant negative indirect effect of the serum vitamin D concentration on HAMD score through TIV in patients with MDD, i.e., the total effect of serum vitamin D concentration on HAMD score was significant (path c = −0.328, SE = 0.141, *P* = 0.025), and was fully mediated by the TIV (indirect effect = −0.1741, SE = 0.0931, 95% CI: −0.3984, −0.0326; path *c*′ = −0.154, SE = 0.148, *P* = 0.303).

### Comparison Between Patients With Severe and Non-Severe Depressive Symptoms

Patients with severe depressive symptoms (*N* = 32, 1,407.01 ± 139.59 cm³) exhibited reduced TIV (two-sample *t* test, *t* = −3.347, *P* = 0.002) compared with patients with non-severe depressive symptoms (*N* = 18, 1,548.68 ± 150.79 cm³). A trend toward decreased serum concentration of vitamin D (two-sample *t* test, *t* = −1.928, *P* = 0.060) was found in patients with severe depressive symptoms (*N* = 30, 42.11 ± 9.97 nmol/L) compared with non-severe depressive symptoms (*N* = 15, 48.90 ± 13.23 nmol/L).

## Discussion

Our main findings are twofold: 1) in patients with MDD, more severe depressive symptoms were related to smaller TIV and lower serum vitamin D concentration, and smaller TIV was related to lower serum vitamin D concentration; 2) TIV was a significant mediator of the association between serum vitamin D concentration and severity of depressive symptoms in the patients.

There are several lines of evidence in support of a plausible association between lower serum vitamin D concentration and the development and clinical manifestations of depression. For example, observational studies have reported that serum concentration of vitamin D was lower among patients with depression when compared to healthy controls (41, 42). Vitamin D has been proven to have neuroprotective effects, and higher serum vitamin D concentrations are related to a reduced risk of depression (19, 43–46). Combined, these previous reports are compatible with our findings that the MDD patients had vitamin D insufficiency or deficiency and lower serum vitamin D concentration was associated with greater severity of depressive symptoms.

A meta-analysis has demonstrated that vitamin D supplementation (≥800 I.U. daily) is somewhat favorable in the management of depression and the effect size is even comparable to that of antidepressant medication (23). There is current evidence that adjunctive use of vitamin D with antidepressants can reduce depressive symptoms more effectively (47). Song et al. have found that vitamin D supplements can predict lower depressive symptoms and reduce cardiac events for MDD patients (48). Wong et al. have revealed that vitamin D supplementation is effective in treating depression and maintaining optimum vitamin D levels is a highly feasible approach to prevent depression (49). However, a meta-analysis did not support the evidence for the efficacy of vitamin D in improving depression among adults (50). Therefore, the eventual impact of a vitamin D supplementation on depressed patients remains uncertain and needs to be investigated further.

The relationship between brain and vitamin D has attracted intense interest from neuroimaging researchers. For instance, previous studies have revealed that vitamin D depletion is associated with smaller brain volume and larger lateral cerebral ventricles (24). In older adults, vitamin D deficiency has be found to be linked to lower hippocampal volume (25) and increased white matter abnormality volume (26). In healthy young women, lower serum vitamin D level has been shown to be associated with greater TIV as well as total cortical gray and cerebral white matter volumes (27). A previous review has highlighted the effects of vitamin D on brain development and adult brain function (51). Therefore, the current finding of a positive correlation between TIV and serum vitamin D concentration in MDD patients not only supports consistent findings in healthy population but also adds to the current knowledge of vitamin D–brain association in a clinical population.

Brain structural studies have revealed widely distributed gray matter volume reductions in patients with MDD, and the affected brain regions mainly comprise orbitofrontal cortex, prefrontal cortex, insula, supplementary motor area, middle temporal gyrus, hippocampus, parahippocampal gyrus and fusiform gyrus, thalamus, cingulate cortex, amygdala, and striatum (28–34, 52, 53). In this study, we found that patients with severe depressive symptoms exhibited reduced TIV compared with those with non-severe depressive symptoms. The inverse correlation between TIV and depression severity implies that lower TIV may render the patients into a risk of experiencing more severe depressive symptoms.

Notably, further mediation analysis indicates that the relationship between serum vitamin D concentration and severity of depressive symptoms can be fully mediated by TIV, which is the most pivotal finding with clinical relevance. On one hand, it provides preliminary evidence that the effects of vitamin D on depression appear to have a neuroanatomical basis, though the relationship between them is complex. On the other hand, TIV can serve as a sensitive and objective imaging biomarker capable of capturing depressive symptom changes along with serum vitamin D level alteration, and thus might be used in a clinical setting to monitor MDD patients’ responses to adjuvant therapy of vitamin D.

Our results need to be interpreted in light of several limitations. First, the fairly modest sample size did not allow us to perform a full analysis of the potential effects of medication and illness duration. Future studies in a larger sample of medication-naive, first-episode patients with MDD are warrant to test the stability of our results. Second, this study is a cross-sectional design, which makes it difficult to draw a conclusion about the causal relationship. Longitudinal studies are required to establish the direction of causality. Finally, this study lacks normal randomized controlled data, which precludes us from exploring the relationship between serum vitamin D concentration and TIV in healthy subjects.

In conclusion, we demonstrated a negative effect of serum vitamin D concentration on severity of depressive symptoms in patients with MDD, which was mediated by TIV. This finding may help to establish TIV as a potential neural biomarker for monitoring responses to adjuvant therapy of vitamin D in patients with MDD.

## Data Availability Statement

The datasets for this manuscript are not publicly available because this is to protect the patient’s privacy. Requests to access the datasets should be directed to 631132271@qq.com.

## Ethics Statement

This study was approved by the ethics committee of The First Affiliated Hospital of Anhui Medical University. All participants provided written informed consent after they had been given a complete description of the study.

## Author Contributions

YYu, JZ, D-mZ, and WZ conceptualized and designed the study. D-mZ and WZ were responsible for conducting the analyses, preparing the first draft of the manuscript, and preparing the manuscript for submission. YYu and JZ were responsible for obtaining funding for the study, supervising the analyses, and editing drafts of the manuscript. WZ, BZ, YZ, CZ, YW, and YYa were responsible for data collection and initial data preprocessing. All authors contributed to and approved the final manuscript.

## Conflict of Interest Statement

The authors declare that the research was conducted in the absence of any commercial or financial relationships that could be construed as a potential conflict of interest.
