# Supplementary material for: The Relationship Between Serum Concentration of Vitamin D, Total Intracranial Volume, and Severity of Depressive Symptoms in Patients With Major Depressive Disorder
Source: Front Psychiatry. 2019 May 9;10:322. doi: 10.3389/fpsyt.2019.00322 (PMC6520644; doi:10.3389/fpsyt.2019.00322)
Supplement: Supplementary file 1 [file Table_1.docx]

**Supplementary Materials**

Table S1. The medication information of 50 patients with major depressive disorder

| Drugs | Number of patients | Types of antidepressants |
| --- | --- | --- |
| Paroxetine | 18 | SSRIs |
| Escitalopram | 8 | SSRIs |
| Mirtazapine | 1 | NaSSA |
| Venlafaxine | 5 | SNRIs |
| Duloxetine | 11 | SNRIs |
| Sertraline | 4 | SNRIs |
| Fluvoxamine | 3 | SSRIs |

Abbreviations: SSRIs, selective serotonin reuptake inhibitors; SNRIs, serotonin and norepinephrine reuptake inhibitors; NaSSA, noradrenergic and specific serotonergic antidepressant.
